# IRMKD: an application of instance relation matrix in plant disease recognition

**DOI:** 10.3389/fbinf.2026.1761574

**Published:** 2026-01-29

**Authors:** Jinqing Huang, Jian Su, Tengfei Cheng

**Affiliations:** 1 School of Computer and Electronic Information, Guangxi University, Nanning, China; 2 Guangxi Science and Technology Evaluation Center, Nanning, China

**Keywords:** convolutional neural network, deep learning, disease identification, Knowledge distillation, model compression

## Abstract

**Background:**

The recognition and prevention of plant diseases is very important to the growth process. At present, neural networks have achieved good results in plant disease identification, but the development of convolutional neural networks has brought a large number of network parameters and long recognition time, which greatly limits its application on devices that lack computing resources.

**Methods:**

To solve this problem, We introduce a novel approach, dubbed instance-relation-matrix based knowledge distillation (IRMKD), that transfers mutual relations of data examples. For concrete realizations of IRMKD, we combine the correlation of the samples with the relationship between the characteristics of the instances and introducing multiple loss functions.

**Results:**

Experimental results show that the proposed method improves educated student models with a significant margin. In particular, for traditional neural networks, our method significantly reduces memory usageand recognition time by an average of 92% and at the same time ensure that the recognition accuracy rate is above 93%, provides a new plant disease recognition method for devices with limited memory and computing resources.

**Conclusion:**

IRMKD can significantly reduce the volume of the model and improve the recognition speed of the model on the premise of slightly reducing the accuracy of the verification set.

## Introduction

Crop diseases have seriously affected the world’s agricultural economy and will cause severe damage to crop yields. Disease identification is the key to predicting agricultural yields, which is of great importance for economic stability and food security in the agricultural sector (Kuzuhara et al., 2020). With the development of deep learning technology, numerous structures or patterns of complex networks are being used to identify diseases. But the enormous computing complexity of these architectures has restricted their use in many downstream applications. In response to this situation, some researchers had proposed different methods of compression of models in recent years. Real et al. (2019) develope an image classifier that exceeds manual design, which makes the neural network model more compact. Deng et al. (2019) propose a compression technique which is not explored in the area of architecture taking into account the decomposition of the tensor. Zhang et al. (2018) jointly train a quantified DNN compatible with bits and its related quantizer to obtain the effect of the compression model. There are also a number of methods to compress network models, including prunin and knowledge distillation (Deng et al., 2020).

As a typical type of model compression and acceleration, knowledge distillation can effectively train small student models from large teacher models (Gou et al., 2021). Knowledge distillation can be divided into the following categories: response-based knowledge distillation, feature-based knowledge distillation, and relation-based knowledge distillation.

Response-based knowledge distillation: Response-based knowledge distillation usually means that the student network responds to the neurons in the last output layer of the teacher model. Its main idea is to directly simulate the final prediction of the teacher model. In recent years, some scholars have further explored response-based knowledge to solve the problem of insufficient information when the ground truth tag is the conditional target (Meng et al., 2019).

Feature-based knowledge distillation: Deep neural network is good at learning multi-level feature representation. Specifically, feature-based knowledge from the middle layer is a good extension of response-based knowledge, especially for the training of thinner and deeper networks. Zhang et al. (2020) propose a new task-based feature distillation (TOFD) method, which is a convolution layer trained by task loss in a data-driven way. Chen et al. (2018) proposed a feature mapping-based knowledge extraction method called knowledge extract with feature maps (KDFM), which improves the efficiency of knowledge extraction by learning feature maps from the teacher network.

Relationship-based knowledge distillation: Both response-based and feature-based knowledge use the output of a specific layer in the teacher model, while relationship-based knowledge distillation further discusses the relationship between different layers or data samples on the basis of the above two methods. Lee and Song. (2019). propose a knowledge distillation method based on multi-head graphs. They explore the data relationship between any two feature graphs in a multi-attention network through graph knowledge. In order to explore the paired clues in the student network and the teacher network, Passalis use the student model to simulate the mutual information flow of the paired clues in the teacher model (Passalis et al., 2020).

At present, the compression method of the above model still has the problem of low compression rate or loss of model accuracy after compression. The common point of these methods is that in the process of knowledge distillation, they only pay attention to the consistency of the instance while ignoring the correlation between the samples (Peng et al., 2019). In fact, the correlation between samples is also very important for classification, because it directly reflects how teachers model the structure of different samples embedded in the feature space. Therefore, we propose a knowledge extraction method based on the relationship between examples. In addition to the widely used instance feature maps, our method also defines three new knowledge types: sample correlation, instance correlation and feature space transformations, and proposes an instance relation matrix (IRM) to model all types of knowledge.

In this paper, combining the plant village disease data set (Hughes and Salathé, 2015) and the complex background data set provided by the Guangxi Academy of Agricultural Sciences, a lightweight convolutional neural network compression method based on knowledge distillation (Hinton et al., 2015) is proposed. The test results in the real environment show that our method can significantly reduce the memory usage of the model while maintaining or slightly reducing the accuracy of the model. In addition, the method we propose is versatile. Whether deploying the model on a cloud server or a local device, this method can improve the recognition speed of the model while reducing memory usage and training overhead. Our main contributions can be summarized in the following three areas:

1. For the first time, we combine the four kinds of knowledge of sample correlation and instance feature, instance relationship and cross-layer feature space transformation to carry out knowledge distillation.
2. For the first time, the concept of instance relation matrix (IRM) is proposed, and the instance relation matrix and its transformation were used to model all types of knowledge. The instance relationship matrix can be represented by the data structure of the three-dimensional array IRM [*i*][*j*][*k*], where *i* and *j* represent the Euclidean distance between the *i*th feature map and the *j*th feature map, and *k* represents the same the *k*th sample in the batch.
3. Introducing multiple loss functions to supervise the training of the student network is used to help students learn different kinds of knowledge stored in IRMs, and then obtain the final loss function $L_{\text{MTK}}$ by weighting, and then prove the superiority of the method through experimental results.

## Materials and methods

### Data preprocessing

**FIGURE 1:**
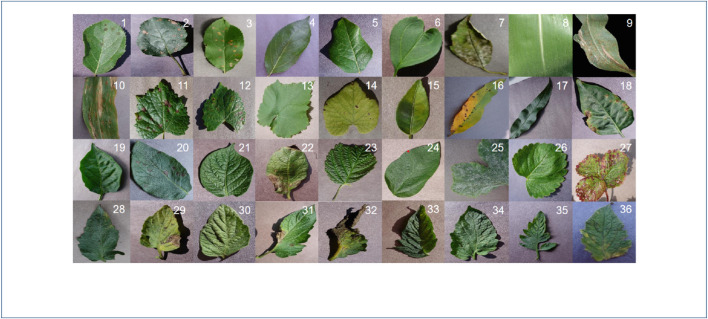
Partial presentation of plant village dataset.

The train set and validation set used in our experiments are based on the Plant Village dataset (Hughes and Salathé, 2015). It contains 82,161 pictures of plant leaves of varying sizes from 24 plants in 55 classes. The data set contains images with clean background and congested background, as shown in Figure 1. Clean background images consist of isolated leaves with uniform backgrounds, while cluttered background images comprise partial or full images of plants taken in a natural background. The number of images in each class ranges from 43 to 6,359. This data set is divided into three different sets. PlantLeaf1 contains 18 classes which contain pictures with a cluttered background. None of the images in this dataset contains laboratory-conditioned images. PlantLeaf2 contains 11 classes, which constitute both clean and cluttered images. Clean background images were used in the training, while cluttered background images were used in the testing of this dataset. PlantLeaf3 consists of 16 classes of 11 plants. These classes contain both clean and cluttered images, whereas the number of images per class varied from 892 to 5,507. This dataset consists of 10 classes of 10 different crop species and 6 classes of tomato plants infected by different diseases. The number of classes and frames for each PlantLeaf data set is detailed in Table 1.

**TABLE 1:** Generated database for training and validation.

Class name	Plant common name	Disease common name	Disease scientific name	Images (number)
C01	Apple	Apple scab	Venturia inaequalis	630
C02	Apple	Apple black rot	Botryosphaeria obtusa	621
C03	Apple	Cedar apple rust	Gymnosporangium juniperi-virginianae	275
C04	Apple	Healthy	—	1,645
C05	Blueberry	Healthy	—	1,502
C06	Cherry	Healthy	—	854
C07	Cherry	Powdery mildew	Podosphaera spp	1,052
C08	Corn	Cercospora leaf spot	Cercospora zeae-maydis	513
C09	Corn	Common rust	Puccinia sorghi	1,192
C10	Corn	Northern leaf blight	Exserohilum turcicum	985
C11	Corn	Healthy	—	1,162
C12	Grape	Black rot	Guignardia bidwellii	1,180
C13	Grape	Black measles	Pc	1,383
C14	Grape	Isariopsis leaf spot	Pseudocercospora vitis	1,076
C15	Grape	Healthy	—	423
C16	Orange	Huanglongbing	Candidatus liberibacter	5,507
C17	Peach	Bacterial spot	Xanthomonas campestris	2,297
C18	Peach	Healthy		360
C19	Pepper	Bacterial spot	Xanthomonas campestris	997
C20	Pepper	Healthy	—	1,478
C21	Potato	Early blight	Alternaria solani	1,000
C22	Potato	Late blight	Phytophthora infestans	1,000
C23	Potato	Healthy	—	152
C24	Raspberry	Healthy	—	371
C25	Soybean	Healthy	—	5,090
C26	Squash	Powdery mildew	Erysiphe cichoracearum	1835
C27	Strawberry	Leaf scorch	Diplocarpon earlianum	1,109
C28	Strawberry	Healthy	—	456
C29	Tomato	Bacterial spot	Xanthomonas campestris	2,127
C30	Tomato	Early blight	Alternaria solani	1,000
C31	Tomato	Late blight	Phytophthora infestans	1909
C32	Tomato	Leaf mold	Fulvia fulva	952
C33	Tomato	Septoria leaf spot	Septoria lycopersici	1771
C34	Tomato	Spider mites	Tetranychus urticae	1,676
C35	Tomato	Target spot	Corynespora cassiicola	1,404
C36	Tomato	Tomato mosaic virus	Tomato mosaic virus	373
C37	Tomato	Yellow leaf curl virus	Begomovirus	5,357
C38	Tomato	Healthy	—	1,591
Total				54,305

To generalize the model and ensure a robust model, these image datasets were augmented using different data augmentation processes, such as flipping, random crops, rotations, shifts, and a combination of these techniques. Data augmentation aims to prevent overfitting by training the model to large data created artificially model.

### Overviewofour knowledge distillation method

**FIGURE 2:**
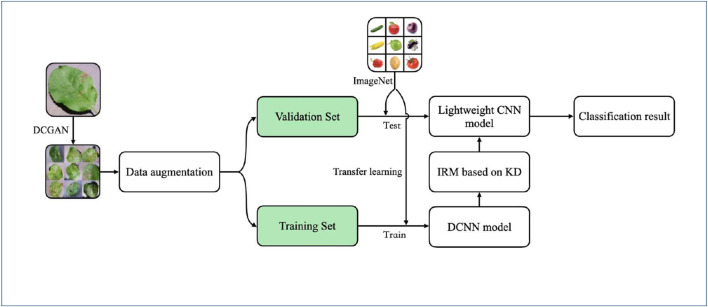
The overview of IRMKD.

In this section, a structured disease identification method and a lightweight neural network reduction method are proposed. The overall design of this study is shown in Figure 2.

Knowledge distillation is first proposed in (Weng and Preneel, 2011) for model compression. The key idea of knowledge distillation is that the soft probability of trained teachers’ network output contains not only class labels, but also more information about data points. For example, if multiple categories of high probability areas signed to an image, it may mean that the image must be located near the decision boundary between these categories. Therefore, forcing students to imitate these probabilities should enable students’ network to absorb some knowledge that teachers has found in the information outside the training label itself.

In the learning process of knowledge distillation (Shorten and Khoshgoftaar), the student model is trained by imitating the output of the teacher model in the same sample. In the traditional ***Softmax*** classifier, given any input image, the model generates a vector $S^{t}\left(x\right)=\left[S_{1}^{t}\left(x\right),S_{2}^{t}\left(x\right),\ldots ,S_{K}^{t}\left(x\right)\right]$ where $S^{t}\left(x\right)$ represents the score corresponding to the *k*th disease. We use ***Softmax*** as the classifier at the end of the neural network to convert the output $S^{t}\left(x\right)$ of the neural network into probability Distribution $p^{t}\left(x\right)$ , as shown in Formula 1.

$$p_{k}^{t}\left(x\right)=\frac{e^{s_{k}^{t}\left(x\right)}}{\sum_{j}e^{s_{j}^{t}\left(x\right)}}$$ (1)

Hinton et al. (2015) proposed that the output of a well-trained teacher model would be infinitely close to the real output of One-Hot coding, which causes useful inter class information to be ignored in the training process, and directly lead to the unsatisfactory training effect of the student model. Therefore, it is necessary to use the temperature scale to “soften” these probabilities, as shown in Formula 2.

$${\tilde{p}}_{k}^{t}\left(x\right)=\frac{e^{s_{k}^{t}\left(x\right)/T}}{\sum_{j}e^{s_{j}^{t}\left(x\right)/T}}$$ (2)

where T > 1 is an adjustable super parameter. By adding the parameter T to classifier, students wound similarly produce a softer classification probability distribution ${\tilde{p}}^{s}\left(x\right)$ , thus preserving the probability relationship between different categories of samples. Compared with the traditional One-Hot coding hard tag as the training target, because the soft target output by the teacher model after the *Soft-Softmax* classifier well retains the probability relationship between different categories of samples, it usually bring better performance. The loss function of students is a linear combination of the typical cross entropy loss function $L_{\mathrm{cls}}$ and the loss function $L_{KD}$ in the process of knowledge distillation (Kim and Rush, 2016), as shown in Formula 3–5.

$$L=\alpha L_{\mathrm{cls}}+\left(1-\alpha \right)L_{KD}$$ (3)

$$L_{KD}=-T^{2}\underset{k}{\sum }{\tilde{p}}_{k}^{t}\left(x\right)\log{\tilde{p}}_{k}^{t}\left(x\right)$$ (4)

$$L_{cls}=-\underset{k}{\sum }q_{k}\log{\tilde{p}}_{k}^{t}\left(x\right)$$ (5)

where T and α are adjustable super parameters. The common choices are T ∈ 3,4,5 and α = ∈ [0.5,0.9], $q_{k}\left(x\right)$ is the real label of the sample.

### Relational knowledge distillation based on IRM

**FIGURE 3:**
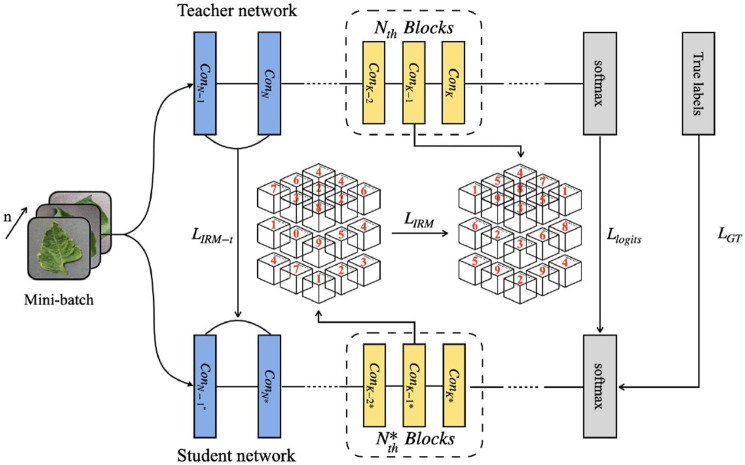
Knowledge distillation structure diagram.

As shown in Figure 3, for multiple DNN layers of the teacher model, an matrix is constructed, where is the number of DNN layers selected, and each element in the matrix represents the Euclidean distance between two characteristic graphs with corresponding subscripts. The matrix provides sufficient and general information about the characteristic distribution, so that the extracted knowledge can guide student networks with different structures. At present, most teacher-student frameworks based on knowledge distillation rely on strong constraints at the instance level.

At the same time, the correlation among multiple samples is also valuable for knowledge distillation. Using the sample correlation, the student model can better learn the relationship between different samples. Therefore, in the process of knowledge distillation, this paper takes n samples as the input of neural network at the same time. Because each sample will generate a m×m matrix, we will finally obtain a n×m×m three-dimensional matrix, which is *IRM*.

Let the input data set of the network be $X=\left\{x_{1},x_{2},\ldots ,x_{n}\right\}$ , n is the sample number of Mini-batch, $C_{i}^{T}\left(x_{i}\right)$ and $C_{i}^{S}\left(x_{i}\right)$ are the output characteristic graphs of the *i*th sample in the *j* layer of teacher and student models respectively, $f_{a.h}^{T}\left(x_{i}\right)$ an mapping $f_{a.h}^{S}\left(x_{i}\right)$ represents the Euclidean distances of the output characteristic graphs of the *g*th and *h*th layers in the teacher student model. Let $F_{i}^{T}$ and $F_{i}^{S}$ represents the set of distances between the *j*th layer and each layer in the *m*th layer in the teacher and student models, respectively via Formulas 6.1, 6.2:

$$F_{j}^{T}\left(x_{i}\right)=\text{matrix }\left(f_{j,1}^{T}\left(x_{i}\right),f_{j,2}^{T}\left(x_{i}\right),\ldots ,f_{j,m}^{T}\left(x_{i}\right)\right)$$ (6.1)

$$F_{j}^{S}\left(x_{i}\right)=\text{matrix }\left(f_{j,1}^{S}\left(x_{i}\right),f_{j,2}^{S}\left(x_{i}\right),\ldots ,f_{j,m}^{S}\left(x_{i}\right)\right)$$ (6.2)

At the same time, the mapping functions are introduced as follows (Formulas 7.1, 7.2):

$$\theta :F\to G\in W^{mxm}$$ (7.1)

$$\varphi :G\to H\in E^{nxmxm}$$ (7.2)

where G is the distance matrix of m×m and H is the three-dimensional matrix of n×m×m , each element in H represents the distance between the characteristic graph of layer $C_{g}$ and layer $C_{h}$ in the input *i*th sample.

The IRM formula can be expressed as follows (Formula 8):

$$L_{IRM}=\Psi \left(\varphi^{T}\left(\chi \right),\varphi^{S}\left(\chi \right)\right)$$ (8)

where Ψ is the loss function as Formula 9:

$$\Psi \left(H^{T},H^{S}\right)=\underset{\begin{array}{c} i\in \left[1,n\right]\\ j,k\in \left[1,m\right] \end{array}}{\sum }{\left\|h_{i,j,k}^{T}-h_{i,j,k}^{T}\right\|}_{2}^{2}$$ (9)

In order to avoid too strict constraints, cross layer feature space transformation is introduced as the third type of knowledge, and an IRM transformation is proposed to model the knowledge. The feature space transformation is a more relaxed description than the dense fitting of teacher’s case features in the middle layer. By combining IRM with IRM transformation, this method has more general, moderate and sufficient knowledge than the existing methods. Finally, two loss functions for IRM and IRM transformation are designed and optimized to improve the performance of the student model. Firstly, the mapping function is defined as follows (Formula 10):

$$\Phi :\chi \to D\in R^{n\times n}$$ (10)

where D is a two-dimensional matrix of n×n , let each element of $D_{i}^{T}$ and $D_{i}^{S}$ represents the distance of the output characteristic graph of the $x_{i}$ sample and the $x_{i^{\prime }}$ sample input in the teacher and student models at the *j*th layer respectively. $L_{IRM-t}$ extracted the transformation knowledge of feature space by calculating the difference change of the feature graph between two layers in the network model, $D_{g}^{T}\left(\chi \right)-D_{h}^{T}\left(\chi \right)$ is the amount of knowledge flow information from layer g to layer h in the teacher network, then *IRM-t* formula can be defined as follows (Formula 11):

$$L_{IRM-t}=\|\left(D_{g}^{T}\left(\chi \right)-D_{h}^{T}\left(\chi \right)\right)-\left(D_{g}^{S}\left(\chi \right)-D_{h}^{S}\left(\chi \right)\right){\|}_{2}^{2}\;$$ (11)

$L_{\mathrm{logits}}$ formula represents the ***Softmax*** loss of teachers’ network output and students’ network output as Formula 12:

$$L_{\mathrm{logits}}={\left\|Y^{T}-Y^{S}\right\|}_{2}^{2}\;$$ (12)

Finally, we define an $L_{MTK}$ loss function is used to train student network, which is based on *IRM-t* transform loss $\left(L_{IRM-t}\right)$ , IRM loss $\left(L_{IRM}\right)$ and Softmax loss $\left(L_{\text{logits }},L_{GT}\right)$ as follows Formula 13.

$$L_{MTK}=\alpha L_{IRM-t}+\left(1-\alpha \right)L_{IRM}+\beta L_{\text{logits }}+\gamma L_{GT}$$ (13)

where $L_{GT}$ is the loss function between the real tag and the student network output, and α , β , γ are the super parameters. Using MTK loss can optimize the student network and obtains three types of knowledge from the teacher network.

### Experiment results and discussion

The hardware environment of this experiment includes Intel i9-10900x (3.20ghz) 10 core 20 thread CPU, NVIDIA geforce RTX2080ti 11 GB * 2 server. The software environment is Windows10 64 bit system, CUDA 9.0, cudnn 7.0, PyCharm 2018.2. The front end and back end of the experimental framework for training model are keras and tensorflow, respectively.

In this paper, 128 × 128 three channel RGB images are used to train 200 epochs. The size of batchis 512, the initial learning rate is 0.1, the momentum is 0.9, the weight depth is $10^{-4}$ , and the number of classes is,the random seed is 2. The experimental results of different knowledge distillation methods under different network structures are shown in Table 2.

**TABLE 2:** Accuracy of different knowledge distillation methods under different network structures.

Teacher net.	Student net.	Baseline	KD	AT	SP	LIRM	${\text{L}}_{\text{MTK}}$	TNA
VGG16 (304.0 MB)	MobileNet (20.8 MB)	91.57%	91.20%	92.27%	93.86%	92.58%	**93.96%**	95.85%
AlexNet (356.6 MB)	MobileNet (20.8 MB)	91.45%	90.89%	93.37%	93.64%	92.46%	**93.87%**	94.46%
GoogleNet (36.6 MB)	MobileNet (20.8 MB)	91.48%	91.14%	91.98%	92.45%	92.67%	**93.32%**	93.28%
ResNet (179.8 MB)	MobileNet (20.8 MB)	91.53%	90.17%	91.67%	91.23%	92.08%	**93.35%**	93.40%

Bold values indicate the highest accuracy.

**FIGURE 4:**
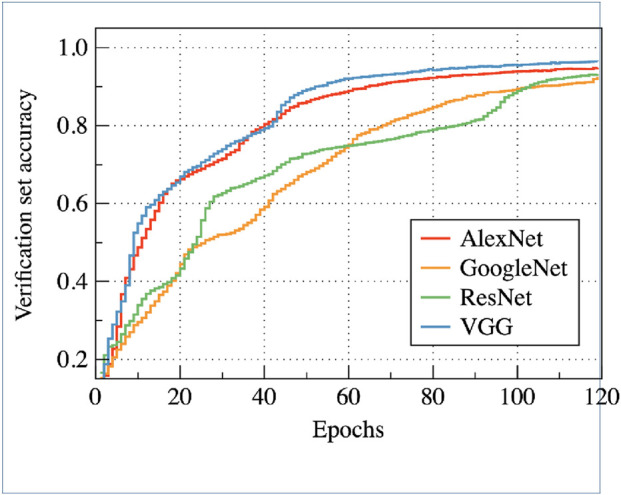
Visual verification set accuracy.

In our experiment, VGG16, AlexNet, GoogleNet and ResNetis used as the teacher’s network structure, and MobileNet is used as the student’s network structure. The teacher column represents the accuracy of the teacher model. First, a teacher model is trained on plant village with 4 different neural network. After 120 iterations, the heighest accuracy of 4 teacher model reaches 95.85%. The column “baseline” indicates the accuracy of the basic student model. The same sample is used to train the student model on MobileNet and the model parameteris only 28.0 MB. Under the same conditions, the accuracy of the model is 91.57%. Figure 4 shows the accuracy change curve of the verification set during the training of four different teacher models.

**FIGURE 5:**
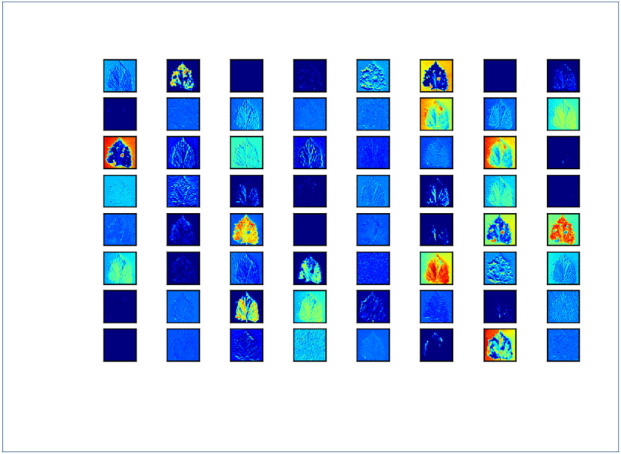
Visualization of disease spot extraction and convolution layer.

The KD column represents the accuracy of the training results after the distillation of basic knowledge. From the table, we can see that the accuracy of the model after the distillation of knowledge has improved compared with the baseline column. AT and SP are the model accuracy of other knowledge distillation methods, $L_{\mathrm{IRM}}$ and $L_{\mathrm{IRM-t}}$ are the model accuracy of the proposed method. It can be seen that using the knowledge distillation method based on the instance relation matrix to transfer the knowledge from the pretrained teacher model VGG16 to the untrained student model MobileNet, the accuracy of the model had been significantly improved, with the highest accuracy of 93.60%. The network structures of teachers and students used are VGG16 (304.0 MB) and MobileNet (28.0M), and the accuracy is only 2.25% different from that of the teacher model. Moreover, the accuracy of the model with multiple loss functions is higher than that with only IRM loss, which proves that the IRM transformation loss $\left(L_{\mathrm{IRM-t}}\right)$ is useful. Figure 5 shows the visualizations of the activation effects of different convolutional layers after IRMKD distillation. As can be seen from the figure, the model has effectively learned to extract and activate the disease spots on the sample leaves.

**FIGURE 6:**
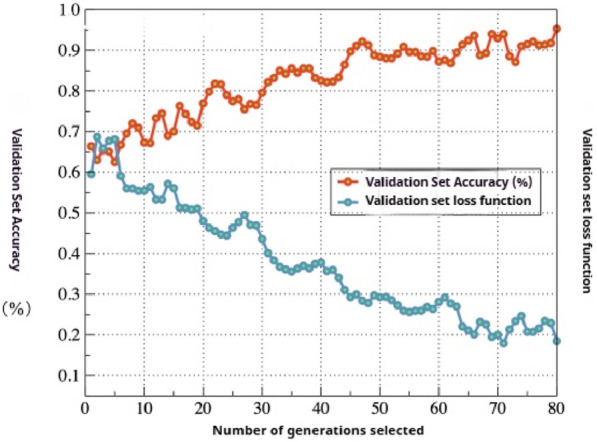
The influence of iterations on Model recognition accuracy and loss function.

To evaluate the performance of the IRMKD method in real-world scenarios, this paper tested the MobileNet model trained using this method on the mango powdery mildew dataset provided by the Plant Protection Research Institute of the Guangxi Academy of Agricultural Sciences. The changes in accuracy and loss function during the model training process are illustrated in Figure 6. From the figure, it can be observed that during the training process, the loss function decreases, and simultaneously, the prediction accuracy on the test set shows an overall increasing trend. Moreover, the model converges rapidly, achieving a good convergence state after 50 iterations, the highest accuracy reached is 95.54%.

This experiment compares VGG16, AlexNet, GoogleNet and Resnet four main neural network structures, and $L_{\mathrm{MTK}}$ model’s verification set accuracy, parameter size in three-channel RGB image of 128 × 128 sizes. The network with higher verification set accuracy and model parameter quantity is taken as teacher model. Otherwise, it is taken as a student model. It can be seen from the table that MobileNet had the least network parameters, and its accuracy is slightly lower than VGG16 and Resnet. Therefore, this paper chose MobileNet as the student model in knowledge distillation. The results show that VGG16 can make the model the highest accuracy in the search space. At the same time, the average accuracy of $L_{\mathrm{MTK}}$ model optimized by knowledge distillation method is 97.62%, which is slightly improved compared with the other four network structures. The distributed MobileNet model has better performance in memory and average recognition time than other networks. The average recognition time is shortened to 0.218 s, and the model size compression is only 19.83MB, in general, the model after knowledge distillation has higher recognition performance, and can meet the requirements of different application scenarios in recognition time and disk occupation. This proves the effectiveness and feasibility of this method.

In order to compare the performance between our proposed method and other plant disease recognition models, we compared the four methods: generalized regression networks (GRNNs) (Wang et al., 2012a), probabilistic neural networks (PNNs), radial basis function (RBF) (Wang et al., 2012b), BP network with PAC (Wang et al., 2012c). The experimental results are shown in Table 3. The test results show that GRNN and PNN have the highest driving accuracy, 97.27%, 98.06% respectively. It can be seen that these four methods have higher recognition accuracy. The accuracy of RBF neural network, PCA and BP network are 96.06% and 95.44%, respectively, which is slightly lower than the previous four methods. Furthermore, although our method is not as accurate as the 6 methods above, it is clearly ahead of the other methods in terms of model parameters and training speed. In addition, the experimental results also show that the accuracy of the model after pre-processing is improved compared to the model without pre-processing, with an average increase of 0.54%.

**TABLE 3:** Comparison with other research methods.

	Original images			Pre-processed images		
Model	Accuracy (%)	Parameters (M)	Time (S/Epoch)	Accuracy (%)	Parameters (M)	Time (S/Epoch)
GRNNs	97.27	165.35	6,714	96.54	165.24	6,102
PNNs	98.06	247.52	7,039	96.17	248.11	6,421
RBF	96.06	214.89	6,273	97.36	215.36	5,926
PCA and BP	95.44	156.16	5,452	98.26	153.23	4,857
IRMKD	93.45	19.8	1,206	94.67	20.2	1,087

Finally, the accuracy of verification set and the change of IRM loss value are visualized. It can be seen that within 100 iterations, with the increase of the number of iterations, the accuracy of verification set increases from about 65% to about 92%, and the loss value of IRM decreases from about 0.6 to about 0.2. The model effect is significantly improved.

## Conclusion

Aiming at the problem of redundancy and low accuracy of traditional plant disease identification methods, this paper proposes a structured compression method based on knowledge distillation. Compared with the classic convolutional neural network model, this method has advantages in performance. Our method can slightly improve the accuracy rate, while greatly reducing the amount of parameters, shortening the recognition time, so that the model can meet higher real-time requirements. This article compares and analyzes the performance of different models. The main experimental results and conclusions obtained are as follows:

1. We compare the performance of different knowledge distillation methods on the Plant Village dataset in 4 network structures of VGG, AlexNet, GoogleNet and ResNet. The experimental results show that the highest accuracy of IRMKD is 93.96% when VGG is used as the teacher model.
2. Compared with other latest knowledge distillation methods, the results show that the Distilled-MobileNet model can slightly improve the accuracy rate while significantly reducing the parameter amount and memory of the model, and speeding up the model recognition speed.
3. In this paper, the model trained by IRMKD method is compared with other state of the art plant disease recognition methods. The experimental results show that IRMKD can significantly reduce the volume of the model and improve the recognition speed of the model on the premise of slightly reducing the accuracy of the verification set.

## Data Availability

The original contributions presented in the study are included in the article/supplementary material, further inquiries can be directed to the corresponding authors.
